# Isolation and characterization of novel bacteriophage vB_KpP_HS106 for *Klebsiella pneumonia* K2 and applications in foods

**DOI:** 10.3389/fmicb.2023.1227147

**Published:** 2023-08-16

**Authors:** Changrong Chen, Zhenxiang Tao, Tengteng Li, Hong Chen, Yong Zhao, Xiaohong Sun

**Affiliations:** ^1^College of Food Science and Technology, Shanghai Ocean University, Shanghai, China; ^2^Shanghai Engineering Research Center of Aquatic-Product Processing and Preservation, Shanghai, China; ^3^Laboratory of Quality and Safety Risk Assessment for Aquatic Products on Storage and Preservation (Shanghai), Ministry of Agriculture and Rural Affairs, Shanghai, China

**Keywords:** antibiotic-resistant, *Klebsiella pneumoniae*, phage, biocontrol, foods

## Abstract

The detection rate of *Klebsiella pneumoniae* in food is increasing, and it has emerged as a food pathogen. Global health is threatened due to the emergence of multidrug-resistant (MDR) and hypervirulent (hv) *K. pneumoniae*. Phages have a promising application as antibacterial agents and have the ability to lyse MDR strains. Hence, phage vB_KpP_HS106 against MDR-hv *K. pneumoniae* strains was isolated from sewage collected from a hospital. It can maintain stable activity at a pH range of 4–12 and a temperature range of 4°C to 50°C. The maximum adsorption rate of phage HS106 was found to be approximately 84.2% at 6 min. One-step growth curve analysis showed that the latent period of HS106 was 10 min and the burst size was approximately 183 PFU/cell. Furthermore, whole genome analysis indicated that the genome of phage HS106 was a double-stranded linear 76,430-bp long DNA molecule with 44% GC content. A total of 95 open reading frames were annotated in the HS106 genome, which did not contain any virulence genes or antibiotic resistance genes. Phage HS106 reduced MDR *K. pneumoniae* in milk by approximately 1.6 log_10_ CFU/mL at 25°C and in chicken by approximately 2 log_10_ CFU/cm^3^ at 25°C. Therefore, vB_KpP_HS106 is a promising alternative to antibiotics for biocontrol against multidrug-resistant *K. pneumoniae* in foods.

## 1. Introduction

*Klebsiella pneumoniae* is a Gram-negative bacterium belonging to the *Enterobacteriaceae* family and has been recognized as the most common nosocomial pathogen (Nazir et al., 2020). It can cause pneumonia, severe infection, sepsis, and other diseases (Guo et al., 2016), which can cause high mortality in immunocompromised humans and newborns (Machado and Bicalho, 2018). In animal husbandry, *K. pneumoniae* causes severe pneumonia, sepsis, meningitis, and mastitis in cattle. Mastitis caused by *Klebsiella* infection is often more severe (Gröhn et al., 2004; Cheng et al., 2018). Studies worldwide have revealed that *K. pneumoniae* can contaminate meat (Veleba et al., 2012) and dairy products (Chen et al., 2009), which contributes to disease and spoilage (Guo et al., 2016). 29 (16%) Carbapenem-resistant *K. pneumoniae* were isolated from 181 chicken samples collected from farms in western Algeria (Chaalal et al., 2020). Drug sensitivity analysis of 857 milk samples collected from dairy farms in Jiangsu Province and Shandong Province showed that MDR *K. pneumoniae* could be detected in 27.4% of milk samples (Yang et al., 2020), which showed that food is one of the vectors for MDR hypervirulent *K. pneumoniae*. The capsule is one of the most important virulence factors of *K. pneumoniae*. Among more than 80 capsule serotypes, K1, K2, K5, K20, K54, and K57 are closely related to various invasive infections in humans, which are called hypervirulent capsular serotypes of *K. pneumoniae*, of which K1 and K2 are the most virulent (Chuang et al., 2006; Paczosa and Mecsas, 2016). Therefore, effective measures to control hypervirulent *K. pneumoniae* are urgently needed. Bacteriophages (phages) have been studied as valuable antimicrobial alternatives for killing drug-resistant bacteria.

Bacteriophages are viruses that specifically infect bacteria. Phages have been studied as valuable antimicrobial alternatives for killing multidrug-resistant bacteria (Hoang Minh et al., 2016). Compared to antibiotics, phages have the ability to self-proliferate on the infection site with higher host specificity (Wittebole et al., 2014; Xu et al., 2015). In addition, the composition of phages is not toxic to eukaryotic cells (Wittebole et al., 2014). *K. pneumoniae* phages are mainly used in preclinical research and clinical treatment. Cao et al. (2015) have shown that phages can cause *K. pneumoniae* to decline sharply in the lungs of mice within 2 h after lung infection. At present, some commercial bacteriophages have been developed, such as SalmoFresh, Salmonelex, Armament, Listex P100, and ListShield, which have been used to inactivate and control different foodborne pathogens in food substrates and biofilms. Phages are currently marketed to target *Salmonella* spp., *Listeria monocytogenes*, *Shigella* spp., and *Escherichia coli* (García-Anaya et al., 2020). Phage is a good candidate for use as a food additive to control *K. pneumoniae* remaining after pasteurization in dairy products.

MDR *K. pneumoniae* have been detected in fruits, vegetables, dairy products, chicken, seafood, and other foods, which cause increasing harm to human health (Koovapra et al., 2016). In previous studies, phages have not been used to control *K. pneumoniae* in foods. In this study, we focus on the use of bacteriophages as biocontrol agents to control *K. pneumoniae*. A lytic phage against multidrug-resistant *K. pneumoniae* strains (K2 capsular type) was isolated from sewage collected from a hospital, and its characteristic features and genome sequence were determined. Finally, the effectiveness of the application of the phage was determined in milk and chicken meat.

## 2. Materials and methods

### 2.1. Bacterial strains and growth conditions

In total, 41 *K. pneumoniae* strains isolated from dairy farms in Shanghai were used in this study. The drug resistance and capsular type of each strain are shown in Table 1 and Supplementary Table 1. The multidrug-resistant (MDR) *K. pneumoniae* 106 (a K2 capsular type) was used as a host bacterium for phage isolation and propagation. All *K. pneumoniae* strains were cultured in 5 mL of LB broth (LB, Land Bridge Technology, Beijing, China) and incubated up to 10^9^ CFU/mL at 37°C with shaking for 4 h.

**Table 1:** The host range of phage vB_KpP_HS106.

Strain	ESBL^a^	Capsular type	Lysis^b^
*K. pneumoniae* 015	−	K62	−
*K. pneumoniae* 111	−		+++
*K. pneumoniae* 001	−		−
*K. pneumoniae* 002	−		++
*K. pneumoniae* 011	−	K50	−
*K. pneumoniae* 048	−		−
*K. pneumoniae* 062	−	K19	++
*K. pneumoniae* 071	−	K50	−
*K. pneumoniae* 072	−	K8	+
*K. pneumoniae* 074	−	K19	+++
*K. pneumoniae* 308	−		−
*K. pneumoniae* 106	+	K2	+++
*K. pneumoniae* 309	+	K62	−
*K. pneumoniae* 311	+	K27	−
*K. pneumoniae* 037	−	K3	+++
*K. pneumoniae* 059	−	K39	+
*K. pneumoniae* 403	−		+
*K. pneumoniae* 102	+		+
*K. pneumoniae* 045	−		+
*K. pneumoniae* 052	−	K39	+++
*K. pneumoniae* 112	+	K2	−
*K. pneumoniae* 206	+		+
*K. pneumoniae* 313	+	K12	−
*K. pneumoniae* 061	+	K31	++
*K. pneumoniae* 211	+	K12	+
*K. pneumoniae* 031	−		+
*K. pneumoniae* 042	−		++
*K. pneumoniae* 204	−		−
*K. pneumoniae* 023	−	K26	+
*K. pneumoniae* 405	−	K16	+
*K. pneumoniae* 036	−	K23	−
*K. pneumoniae* 077	−		++
*K. pneumoniae* 304	−		+
*K. pneumoniae* 058	+		++
*K. pneumoniae* 103	+	K41	−
*K. pneumoniae* 411	+		−
*K. pneumoniae* 413	+		−
*K. pneumoniae* 006	−	K39	+
*K. pneumoniae* 021	−	K26	+
*K. pneumoniae* 060	−	K28	−
*K. pneumoniae* 208	−	K52	++

a ESBL, Extended-Spectrum-β-lactamase.

b Lytic, +++, complete lysis; ++, lysis; +, turbid lysis; −, no plaques.

### 2.2. Isolation and purification of bacteriophage

*K. pneumoniae* 106 was used as the bacterial host for phage isolation, using the method described previously (Cao et al., 2021). Briefly, sewage (40 mL) collected from Shanghai NO.6 People Hospital was mixed in 40 mL of LB, then 400 μL of 1 M CaCl_2_ (Sangon Biotech, Shanghai, China) was added to enhance the phage adsorption rate and inoculated with 1 mL of *K. pneumoniae* 106 culture (10^9^ CFU/mL). The mixture was incubated at 37°C for 24 h with shaking at 120 rpm. After incubation, the bacteria were removed by centrifugation (8,000× *g* for 10 min at 4°C) (5424, Eppendorf AG 22331, Hamburg, Germany), and the supernatant was filtered through 0.22 μm filters (Millipore, Billerica, MA, USA). The filtrate was collected and used in a spot test to detect the presence of phages. The phages were purified at least three times to obtain a pure phage using a double-layer agar method (Huang et al., 2018). The purified phages were stored at 4°C for further experiments.

### 2.3. Phage host range determination

As the phage host range is an essential factor for phage therapy and decolonization, we performed spot assays to determine the host range of the phage against 41 MDR *K. pneumoniae* strains using the method described previously (Bao et al., 2019). Briefly, 10 μL of suspension containing phage particles (10^8^ PFU/mL) was dropped onto lawn cultures of 41 MDR *K. pneumoniae* strains (Table 1). After overnight incubation at 37°C, the plates were observed for the presence of plaques on the bacterial lawns.

### 2.4. Transmission electron microscopy of the phage

Cesium chloride density gradient centrifugation was used to purify the enriched phages. Transmission electron microscopy (TEM) was used to observe the morphology of the phage according to Yuan et al. (2015) method. First, 20 μL of phage solution (10^9^ PFU/mL) was added dropwise to copper mesh and fixed for 10 min, and the residual liquid was absorbed by filter paper. Then, 2% phosphotungstic acid was added to stain for 2 min. The sample was air-dried and observed by transmission electron microscope (Philips, Eindhoven, The Netherlands).

### 2.5. Temperature and pH tolerance of the phage

The temperature stability of the phage was tested after treatment in a water bath at 4°C, 25°C, 37°C, 50°C, 60°C,70°C, and 80°C for 1 h using the double-layer agar plate. In order to detect the stability of the phage at different pH values, SM buffer with different pH values (2–13) were prepared using 1 M HCl or 1 M NaOH. At each pH value, 100 μL of the phage and 900 μL SM buffer were mixed. After incubation at 37°C for 1 h, the phage titer was determined using the double-layer agar method.

### 2.6. Optimal multiplicity of infection (MOI) assay

The MOI of the phage was determined according to the method described by Li et al. with some modifications (Li et al., 2020). Briefly, the concentration of the phage was adjusted to 10^9^, 10^8^, 10^7^, 10^6^, 10^5^, and 10^4^ PFU/mL, then the host strain was mixed with MOIs of 100, 10, 1, 0.1, 0.01, 0.001, and 0.0001, respectively. After the mixture was incubated for 4 h at 37°C, the phage titer was determined by the double-layer agar method. The MOI that generated the highest phage titer was considered as the optimal MOI (Feng et al., 2021).

### 2.7. Phage adsorption rate and one-step growth curve

In order to determine the adsorption rate of phage to host bacteria, phage was mixed with *K. pneumoniae* at the optimal MOI and incubated at 37°C. For a period of 10 min, 100 μL of the mixture was taken at 1 min intervals and then diluted with 0.9 mL LB. The mixture was centrifuged (12,000 × g, 5 min), and the supernatant containing unabsorbed phages was diluted and counted using the double-layer agar plate method. The adsorption rate was expressed by calculating the percentage of free phage in the culture system. The one-step growth curve of the phage was determined using the method described previously with minor modifications (Wang et al., 2019). Briefly, 8 mL of *K. pneumoniae* 106 culture (10^9^ CFU/mL) was centrifuged (5,000× *g*, 5 min), the supernatant was discarded, and the precipitate was resuspended in 8 mL of SM buffer. The phage at the optimal MOI was added to the suspension and incubated at 37°C for 6 min. The mixture was then centrifuged at 12,000× *g* for 2 min to remove unabsorbed phages. The pellet was resuspended in 10 mL LB broth and incubated at 37°C. Samples were taken at 10 min intervals during the 100 min period then diluted and counted using the double-layer agar plate method.

### 2.8. Phage genome extraction and sequencing

Phage DNA was extracted using the phenol/chloroform extraction method (Yuan et al., 2012). Phage DNA libraries were prepared using the Whole Genome Shotgun strategy (WGS) and then sequenced using Illumina NovaSeq. The Sequencing results with the splice sequences removed were first assembled from scratch using A5-miseq v20160825 (Coil et al., 2015) and SPAdesv3.12.0 (Bankevich et al., 2012) to construct contigs. Collinearity analysis was performed on contig, scaffold and the published sequences in GenBank to determine the position of contigs, and fill the gaps between contigs by Mummerv 3.1 (Delcher et al., 2003). The final sequence was assembled by Pilonv1.18 (Walker et al., 2014). The GeneMarkS interface^1^ was used to predict open reading frames (ORFs) (Blake and Cohen, 2001). The non-redundant database (NR) of the National Center for Biotechnology Information (NCBI) was used to annotate protein-coding genes functionally. The tRNA genes were predicted using the tRNAscan-SE (Kortright et al., 2019). Antibiotic resistance genes and virulence genes were compared in Antibiotic Resistance Database (ARDB^2^) and Virulence Factor Database (VFDB^3^) (Kleinheinz et al., 2014). GCview server (Stothard and Wishart, 2005) was used to draw the gene map of the phage. The comparison of genome sequences between the phage isolated in this study and its most similar genome was visualized using the EasyFig visualization tool (Sullivan et al., 2011). Phylogenetic trees of the phage based on the major capsid protein and terminase large subunit were also analyzed using MEGA 7 (Kumar et al., 2018) with 1,000 bootstrap replications.

### 2.9. Inhibition effect of the phage against *Klebsiella pneumonia*

#### 2.9.1. Inhibition effect of the phage against *Klebsiella pneumonia* in LB broth

The inhibition ability of the phage against MDR *K. pneumoniae* 106 in LB broth was detected in 100-well microtiter plates by the method previously described (Alves et al., 2014). Briefly, 100 μL of *K. pneumoniae* 106 was added to 100-well microtiter plates, and the same volume of phage suspension was added to 100-well plates, to which bacteria had been added at 1, 10, and 100 of MOI. As a control, 100 μL of SM buffer was added. The absorbance at 600 nm was measured at 1 h intervals within 12 h by a Bioscreen C Microbiology Reader (Oy Growth Curves Ab Ltd., Helsinki, Finland).

#### 2.9.2. Inhibition effect of the phage against *Klebsiella pneumonia* in milk

To investigate the inhibition effect of the phage on *K.pneumoniae* in milk, sterile skim milk purchased from the Lotus supermarket in Shanghai, China was used as the medium for *K. pneumoniae* according to the method previously described (Noor Mohammadi et al., 2021). Briefly, 50 μL of *K.pneumoniae* 106 was inoculated into 5 mL of skim milk at a final concentration of 10^4^ CFU/mL, and 50 μL of the phage (10^5^, 10^6^ PFU/mL, MOI of 10, 100) was added to the contaminated milk. SM buffer was added instead of phage suspension as the control group. The mixture was incubated at 4°C and 25°C for 24 h. At 0, 3, 6, 12, and 24 h, 100 μL of the mixture was taken for counting.

#### 2.9.3. Inhibition effect of the phage against *Klebsiella pneumonia* in chicken

The inhibition effect of the phage against *K. pneumoniae* in chicken was assessed according to the method with some modifications (Noor Mohammadi et al., 2021). Chicken meat samples were purchased from the Lotus supermarket in Shanghai, China. The chicken meat was cut into pieces (1 cm × 1 cm × 1 cm) and put into a sterile container containing 0.2% NaCIO for 30 min then washed three times with sterile water. Each piece of the chicken meat was inoculated with 20 μL of *K. pneumoniae* suspension to approximately 1 × 10^4^ CFU/cm^3^. Then, 20 μL of phage solution was added to the final titer of 1 × 10^5^, 1 × 10^6^ PFU/cm^3^. SM buffer instead of phage solution was used as the control. Samples were incubated at 4°C and 25°C for 24 h. The treated chicken samples were placed in 5 mL of PBS buffer and shaken at 160 rpm/min for 5 min. The suspension was centrifuged at 8000 rpm/min for 1 min. The precipitate was resuspended with 1 mL of PBS buffer to remove the phage. The viable bacteria were counted by multiplicative dilution of the bacterial suspension.

### 2.10. Statistical analysis

All experiments in this study were repeated three times. The data were expressed as mean ± standard deviation (SD) and the differences were analyzed with two-way ANOVA using GraphPad Prism 9.0. Differences were considered statistically significant at *p* < 0.05.

## 3. Results

### 3.1. Isolation and general features of bacteriophage

**Figure 1:**
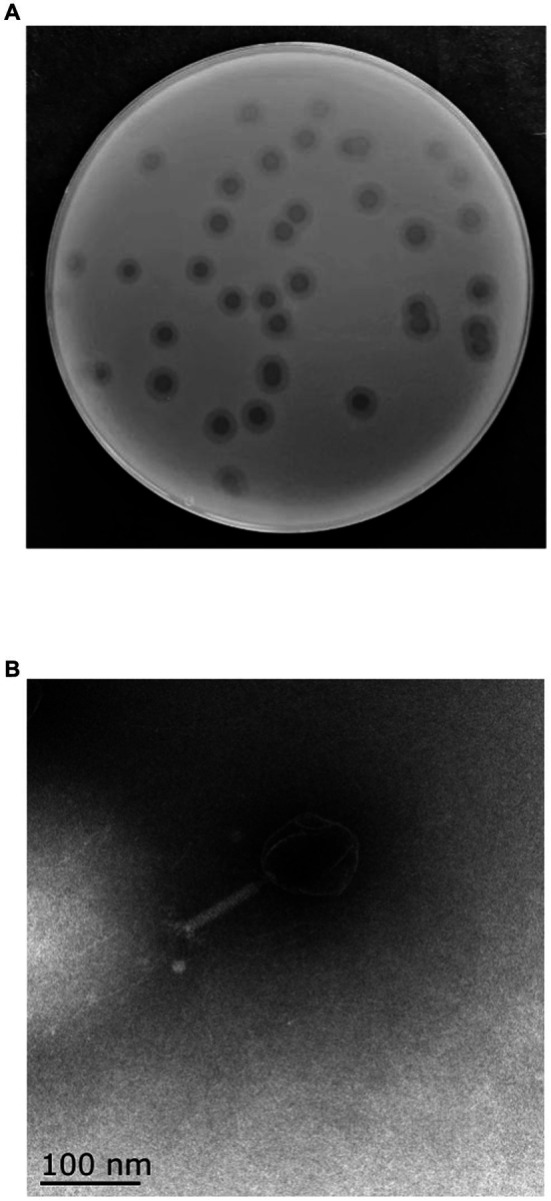
Morphology of phage vB_KpP_HS106. **(A)** Phage vB_KpP_HS106 plaques. **(B)** Transmission electron micrograph of phage vB_KpP_HS106.

One phage was isolated from sewage collected from ShangHai NO.6 People Hospital using MDR *K. pneumoniae* 106 as a host and was designated as vB_KpP_HS106 (phage HS106). The plaque morphology of phage HS106 is shown in Figure 1A. Phage HS106 produced large plaques (diameter, 6.8 mm) with a halo zone (diameter, 5.9 mm) after 12 h. The morphology of phage HS106 observed by TEM is shown in Figure 1B. Phage HS106 had an icosahedral head (diameter about 100 nm) and a long tail (diameter about 100 nm). It can be classified as part of the *Schitoviridae* family according to the demarcation criteria of the International Committee on Taxonomy of Viruses (ICTV) (Adriaenssens et al., 2020).

### 3.2. Host range and the optimal MOI

**Figure 2:**
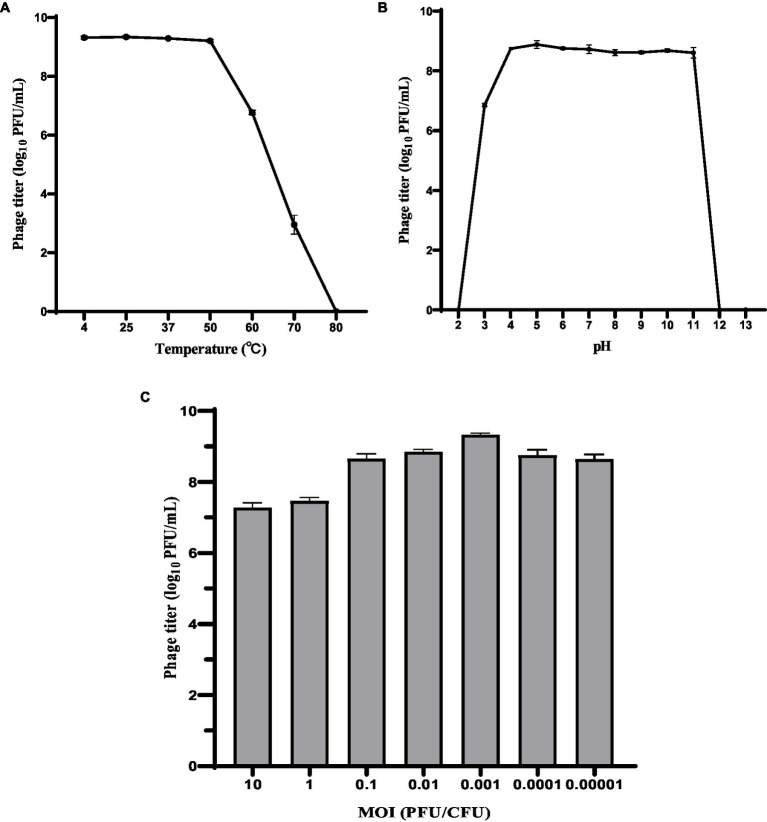
Biological characteristics of phage HS106. Stability of phage vB_KpP_HS106 at various **(A)** temperatures and **(B)** pH values. **(C)** Optimal MOI of phage HS106. The data represent the mean ± SD (*n* = 3).

The host range of phage HS106 is shown in Table 1. Among the 41 *K.pneumoniae* strains used in this study, 26 could be lysed by phage HS106, which had the ability to lyse multiple capsular serotypes. Phage HS106 could lyse MDR *K.pneumoniae,* including *K.pneumoniae* 211, *K.pneumoniae* 061, and *K.pneumoniae* 102. At an MOI of 0.001, the phage HS106 titer was the highest (Figure 2C). Thus, the optimal MOI of phage HS106 was 0.001.

### 3.3. Phage temperature and pH stability

To investigate the activity in different environmental conditions, phage HS106 titer was determined at different temperatures and pH values. As shown in Figure 2A, phage titers were stable at approximately 10^9^ PFU/ml after 1 h of treatment at 4°C to 50°C. The phage titer decreased to 10^7^ PFU/ml and 10^3^ PFU/ml after 1 h of exposure at 60°C and 70°C, respectively. When phage HS106 was incubated at 80°C for 1 h, no phage was detectable. As shown in Figure 2B, there was no noticeable reduction after 1 h of incubation at pH 4 to 11. However, when phage HS106 was incubated at pH 3 for 1 h, the titer of the phage decreased to 10^3^ PFU/ml. Phage HS106 had no infection ability after exposure to pH 2, pH 12, or pH 13 for 1 h. In brief, phage HS106 had wide tolerance to temperatures and pH.

### 3.4. Adsorption rate and one-step growth curves

**Figure 3:**
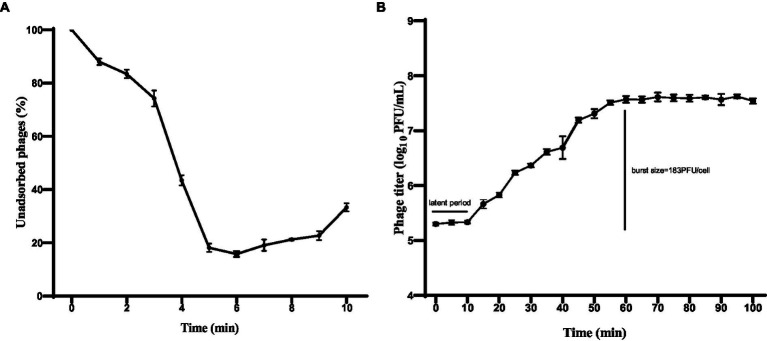
Characteristics of phage vB_KpP_HS106. **(A)** Adsorption assay of phage vB_KpP_HS106; **(B)** One-step growth curve of phage vB_KpP_HS106.

The adsorption rate of phage HS106 is shown in Figure 3A. After phage HS106 was added, the number of unadsorbed phages decreased before 6 min. At 6 min, 84.2% of the phage was adsorbed to the host cells, which was the maximum adsorption rate. To further investigate the proliferative capacity of phage HS106 after infecting the host cell, the one-step growth curve was determined. As shown in Figure 3B, the latent period of phage HS106 was 10 min and the lysis period was 50 min. The burst size was approximately 183 PFU/cell.

### 3.5. Genome characterization of phage HS106

**Figure 4:**
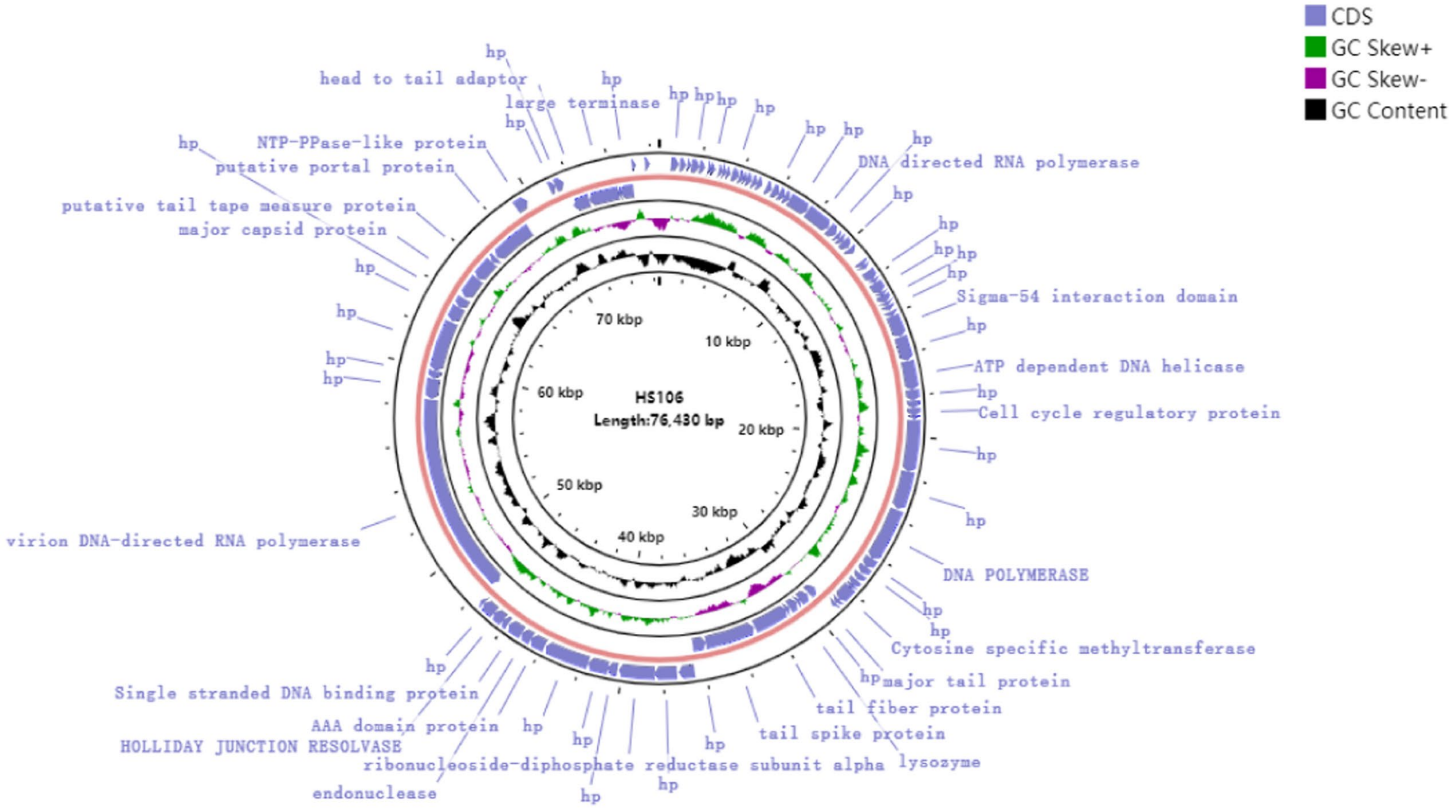
Circular genome annotation of phage vB_KpP_HS106. The inner rings show genome location, GC skew + (green) and − (purple) and GC content (black). The most external rings show identified open reading frames.

Through assembly and annotation to sequencing data, the phage HS106 genome map is shown in Figure 4. The phage HS106 genome consists of a 76,430 bp linear double-stranded DNA, with a G + C content of 44.0%. The whole genome sequence of phage HS106 was uploaded to the GenBank database with login number OP764672.1. A total of 95 open reading frames (ORFs) were identified with 73 ORFs on the positive strand and 22 ORFs on the negative strand. The 27 ORFs (28.4%) were assigned functions, which were predicted to encode functional proteins associated with DNA replication recombination and regulation, metabolism, phage structure and packaging proteins, and host cell lysis. The DNA replication recombination and regulation module include 7 ORFs: DNA polymerase, RNA polymerase, ATP-dependent DNA helicase, and Holliday junction resolvase. The metabolism module includes 6 ORFs: NTP-PPase-like protein, cell cycle regulatory protein, cytosine-specific methyltransferase, ribonucleoside-diphosphate reductase subunit alpha, and endonuclease. The phage structure and packaging proteins module includes 11 ORFs: major tail protein, putative portal protein, terminase large subunit, and PWWP domain-containing protein. Only 3 ORFs belong to the host lysis module (tail fiber/spick protein, Lysozyme). Sequence analysis showed that phage HS106 did not contain any lysogenic factors, which indicates that it belongs to lytic phages. Genome sequencing and analysis indicated that phage HS106 did not contain virulence factor genes or antibiotic resistance genes.

### 3.6. Phylogenetic and comparative genomic analysis of phage HS106

**Figure 5:**
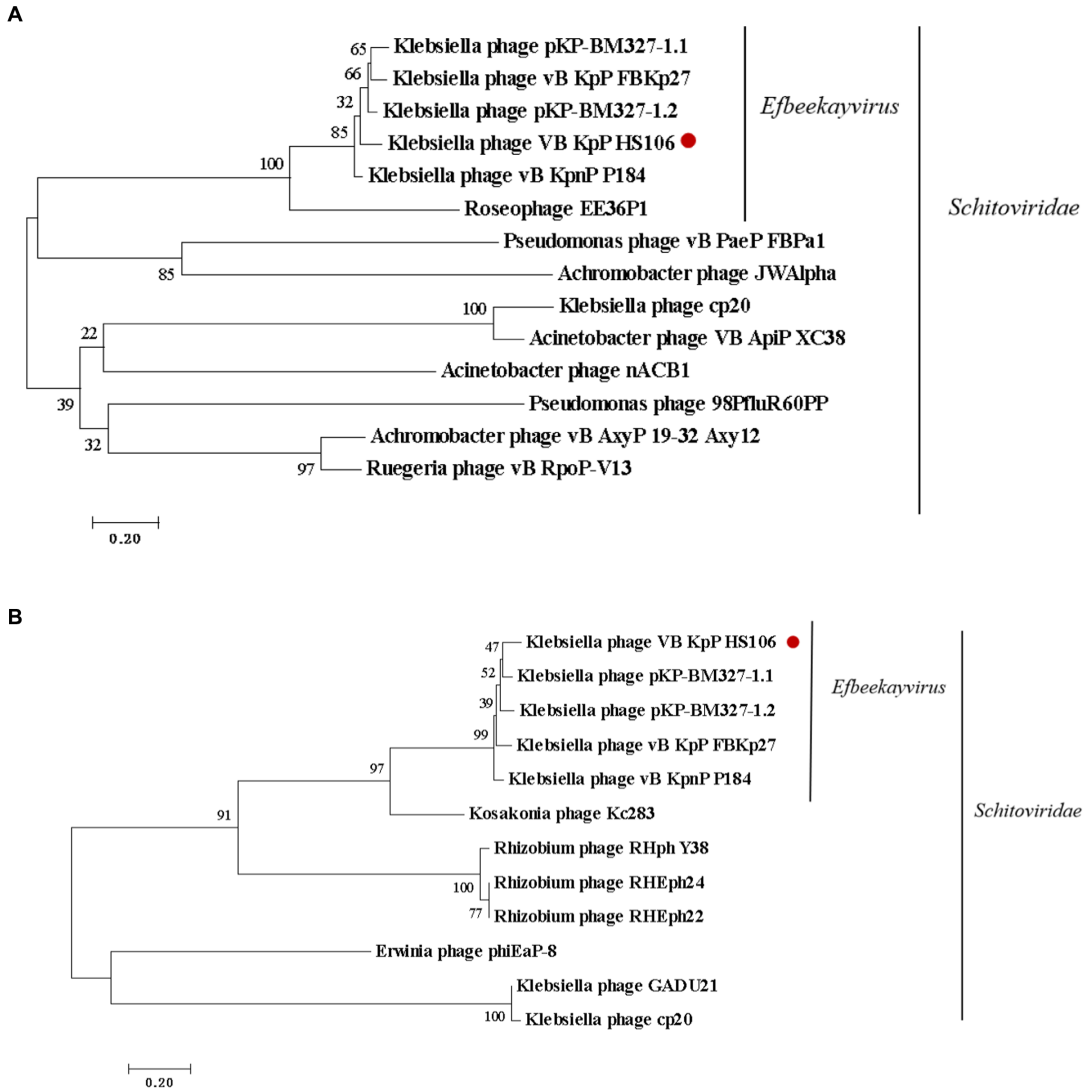
Phylogenetic analyses of phage vB_KpP_HS106. Phylogenetic analyses of selected phages and phages of the *Efbeekayvirus* genus based on the protein sequence of **(A)** major capsid protein and **(B)** terminase large subunit.

Compared with NCBI BLASTn, phage HS106 shared the highest nucleotide identity with *Klebsiella* phage vB_KpnP_P184 (accession:NC_055919.1). The sequence identity between vB_KpP_HS106 and vB_KpnP_P184 was 83.86% (coverage (88.00%) × identity (95.30%) = 83.86%). Comparative genomic analysis using Easyfig software showed that phage HS106 and phage P184 contain similar ORFs. However, there were differences in the tail proteins (ORF60, ORF61), Thymidylate synthase complementing protein (ORF64), and some hypothetical proteins (Supplementary Figure S1). To investigate the relationship between phage HS106 and other phages belonging to the *Schitoviridae* family, a phylogenetic tree based on the major capsid protein and terminal enzyme large subunit is shown in Figure 5. Phage HS106 is classified as a member of the *Efbeekayvirus* genus of the *Schitoviridae* family.

### 3.7. Inhibition effect of phage HS106 against *Klebsiella pneumonia*

#### 3.7.1. Inhibition effect of phage HS106 against *Klebsiella pneumonia* in LB broth

**Figure 6:**
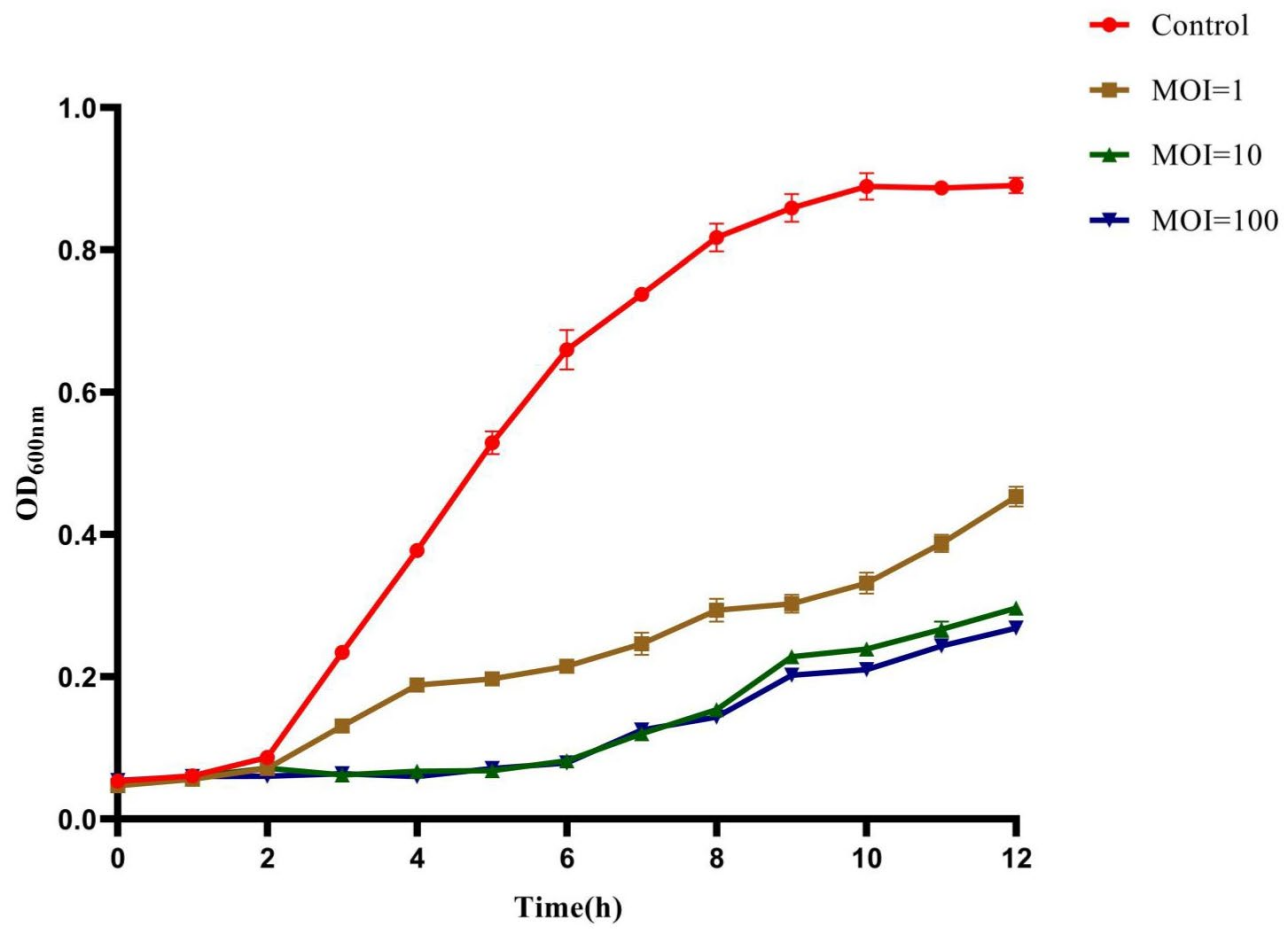
Bacteriolytic activity of phage vB_KpP_HS106 at different MOIs against *K. pneumoniae* in LB broth.

The inhibition effect of phage HS106 on *K. pneumoniae* was evaluated *in vitro*. As shown in Figure 6, during the phage infection for 6 h, the OD_600_ values of phage HS106 treatment groups with MOI of 1, 10, and 100 were less than 0.2. The OD_600_ values of phage HS106 treatment groups with MOI of 10 and 100 were always less than the treatment group with MOI of 1 after incubation for 2 h. After 8 h, the OD_600_ value of the treatment group with high MOI was less than low MOI.

#### 3.7.2. Inhibition effect of phage HS106 against *Klebsiella pneumonia* in milk

**Figure 7:**
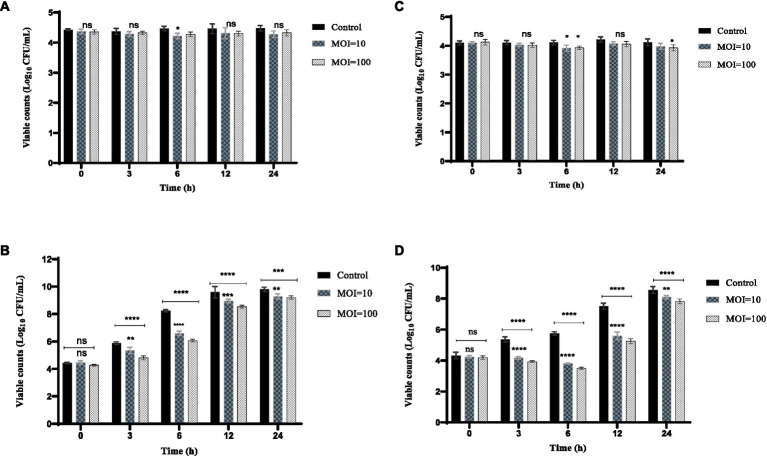
Effects of phage vB_KpP_HS106 on the viability of *K. pneumoniae* 106 in foods. Milk at **(A)** 4°C and **(B)** 25°C. Chicken meat at **(C)** 4°C and **(D)** 25°C. All the values were tabulated as mean ± SD and a significant difference between variations was denoted by asterisks using two-way ANOVA. ****Significant at *p* < 0.0001; ***Significant at *p* < 0.001; **Significant at *p* < 0.01; *Significant at *p* < 0.05; ns, not significant at *p* > 0.05.

The inhibition effect of phage HS106 on *K. pneumoniae* 106 in milk is shown in Figure 7. At 4°C, the amount of *K. pneumoniae* 106 was not significantly different from the control group within 24 h (Figure 7A). The phage HS106 treated group decreased by 1.6 log_10_ CFU/mL at 6 h compared with the control group at 25°C (Figure 7B). After 6 h, the bacterial number in the treated group increased by approximately 2 log_10_ CFU/mL. The results indicated that the antibacterial effect of phage HS106 with high MOI was better than those with low MOI.

#### 3.7.3. Inhibition effect of phage HS106 against *Klebsiella pneumonia* in chicken meat

The inhibition effect of phage HS106 on *K. pneumoniae* 106 in chicken meat is shown in Figure 7. The amount of *K. pneumoniae* 106 was not significantly reduced with phage HS106 treatment for MOI of 10 and 100 compared to the control. At 4°C, the phage did not have a significantly inhibiting effect on *K. pneumoniae* (Figure 7C). At 25°C, the amount of *K. pneumoniae* 106 was reduced by approximately 2 log_10_ CFU/cm^3^ compared with the control group at 6 h. The number of bacteria increased by 4 log_10_ CFU/cm^3^ after 6 h (Figure 7D). The results showed that the antibacterial effect of phage HS106 with high MOI was better than those with low MOI.

## 4. Discussion

The invention of antibiotics solved the problem of bacterial infections and saved countless lives (Kumar et al., 2012). At present, the drug resistance of *K. pneumoniae* is becoming increasingly serious (Ferreira et al., 2019). Phages have emerged as a viable alternative to antibiotics in the face of MDR *K. pneumoniae* (Domingo-Calap et al., 2016). In this study, a lytic phage, phage HS106, was isolated from sewage.

The host range of a phage is one of the most important criteria for biological control. The wider the host range of a phage, the wider its application prospects (Koovapra et al., 2016). In this study, 63% (26/41) of *K. pneumoniae* could be lysed by phage HS106. The 26 *K. pneumoniae* strains included multiple capsular serotypes. Phage HS106 had a wide host range and good application prospects. Phages with a wide host range may be not only related to tail fiber/spick protein and lysozyme but also to proteins of unknown function. Further exploration is required to confirm the presence of unknown proteins capable of lysing *Klebsiella* strains of other capsule serotypes (Pan et al., 2017). The ability of phages to survive in extreme, harsh environments is also an evaluation criterion for phage applications. In this study, phage HS106 maintained stable activity at pH 4-pH 12 and 4–50°C. Compared with the *Klebsiella* phage reported previously (Peng et al., 2020; Zhang et al., 2021), phage HS106 had better tolerance to an extreme, harsh environment, which indicates that it has better activity as a biocontrol agent for application in food.

It can be less costly to reproduce a large number of bacteriophages with a smaller MOI and a shorter incubation period (Abedon, 1989). In this study, Phage HS106 was determined to have an optimal MOI of 0.001 and an incubation period of 10 min, which was considerably better than other *K. pneumoniae* phages, including vB_KpnP_Bp5 (Zhang et al., 2021), vB_KleS-HSE3 (Peng et al., 2020), and vB_KpP_TUN1 (Eckstein et al., 2021), etc. The burst size of phage HS106 was larger than phage TUN1 (Eckstein et al., 2021). In brief, phage HS106 has a smaller optimal MOI, a shorter latent period, and a larger burst size, which indicates higher growth efficiency.

The genome size of *K. pneumoniae* phage is in the range of 19,260–346,602 bp (Peng et al., 2020). Phage HS106 has a 76,430 bp linear double-stranded DNA, which is in the range of *K. pneumoniae* phages. Phage HS106 was classified as *Schitoviridae* by genomic comparison. Genome annotation of phage HS106 predicted four modules: DNA replication recombination and regulation, metabolism, phage structure and packaging proteins, and host cell lysis. ORF61 and ORF62 were annotated as tail fiber/spick protein in the HS106 genome, which shared 84.42% identity with the *Klebsiella* phage vB_KpnP_P184 tail fiber/spick protein sequence (GenBank: YP_010114884.1). Tail fiber/spick protein had a pectate domain, which was a depolymerase used to depolymerize bacterial exopolysaccharides. ORF59 was annotated as lysozyme in the HS106 genome, which shared 96.82% identity with the *Caudoviricetes* sp. Lysozyme protein sequence (GenBank: DAQ70617.1). Lysozyme is inhibited from degrading the peptidoglycan by being tethered to the inner membrane and by having its catalytic domain locked in an inactive conformation either covalently or non-covalently (Young, 2014). Pertics confirmed the potential of phages and lysozyme as candidates for antimicrobial agents (Pertics et al., 2021). According to the classification system of the ICTV, the main species classification standard for bacterial and archaeal viruses is 95% genome similarity (Adriaenssens et al., 2020). Phage HS106 has 83.9% DNA sequence identity with vB_KpnP_P184. Therefore, the phage HS106 isolated in this study was a new species of the genus *Efbeekayvirus.* Genome sequencing and analysis indicated that phage HS106 did not contain any virulence factor genes or antibiotic resistance genes. Therefore, phage HS106 could be safely applied in the food hygiene industry.

In recent years, *K. pneumoniae* has been frequently isolated in foods such as fruits, vegetables, dairy products, chicken, and seafood. The risk caused by Extended-Spectrumβ-lactamase (ESBLs) *K.pneumoniae* is increasing (Koovapra et al., 2016). Phages only exclusively search for the corresponding host bacteria, which are harmless to other bacteria in the intestine. Therefore, they do not interfere with the normal flora and metabolism of the body, and the biological body itself can also clear phages in the body through the immune system (Hagens and Loessner, 2007). Phages have been applied to food products to inhibit pathogens, for example, it has been reported that phage ZPAH7 could reduce *A. hydrophila* by 1.2 log_10_ CFU/m^2^ on lettuce (Islam et al., 2021). In this study, we focused on the use of phage HS106 as a biocontrol agent to control *K. pneumoniae* in food (milk and chicken). At 25°C, phage HS106 reduced MDR-*K. pneumoniae* by 1.6 log_10_ CFU/mL in milk and 2 log_10_ CFU/cm^3^ in chicken. Therefore, phage HS106 is a good candidate for use as a food additive to control *K. pneumoniae* remaining after pasteurization in dairy products. However, in milk and chicken meat, viable counts of *K. pneumoniae* increased after 12 h at 24°C, even in the presence of phage HS106 at an MOI of 100, suggesting that bacteria developed phage resistance (data not shown). The use of a phage cocktail or food additives combined with the phage could be an alternative approach to prevent phage resistance (Soni et al., 2012; Liu et al., 2015).

In conclusion, we isolated a novel lytic phage, vB_KpP_HS106, with high-efficient lysis activity and a wide lysis spectrum. Phage HS106 was found to have a good tolerance to extreme environments. Genome analysis indicated that phage HS106 is a new species of the *Efbeekayvirus* genus of the *Schitoviridae* family. In addition, this phage does not carry any virulence genes or antibiotic resistance genes. It can effectively reduce MDR *K. pneumoniae* in milk and chicken. Therefore, phage HS106 has prospects as a biocontrol agent used to inhibit MDR *K. pneumoniae* in foods.

## Data availability statement

The original contributions presented in the study are publicly available. This data can be found here: National Center for Biotechnology Information (NCBI) GenBank, https://www.ncbi.nlm.nih.gov/genbank/, OP764672.1.

## Author contributions

XS: methodology. CC: software, data curation and writing—original draft preparation. HC, TL, and ZT: investigation. XS and TL: writing—review and editing. YZ: supervision. XS: project administration. All authors have read and agreed to the published version of the manuscript.

## Funding

This work was supported by Shanghai Agriculture Applied Technology Development Program, China (Grant No. 2019-02-08-00-10-F01149).

## Conflict of interest

The authors declare that the research was conducted in the absence of any commercial or financial relationships that could be construed as a potential conflict of interest.

## Publisher’s note

All claims expressed in this article are solely those of the authors and do not necessarily represent those of their affiliated organizations, or those of the publisher, the editors and the reviewers. Any product that may be evaluated in this article, or claim that may be made by its manufacturer, is not guaranteed or endorsed by the publisher.

## Footnotes

^1^ http://exon.gatech.edu/GeneMark/

^2^ https://card.mcmaster.ca/analyze/rgi

^3^ http://www.mgc.ac.cn/VFs/
