# Supplementary material for: Isolation and characterization of novel bacteriophage vB_KpP_HS106 for Klebsiella pneumonia K2 and applications in foods
Source: Front Microbiol. 2023 Aug 16;14:1227147. doi: 10.3389/fmicb.2023.1227147 (PMC10466807; doi:10.3389/fmicb.2023.1227147)
Supplement: Supplementary file 1 [file Image_1.pdf]

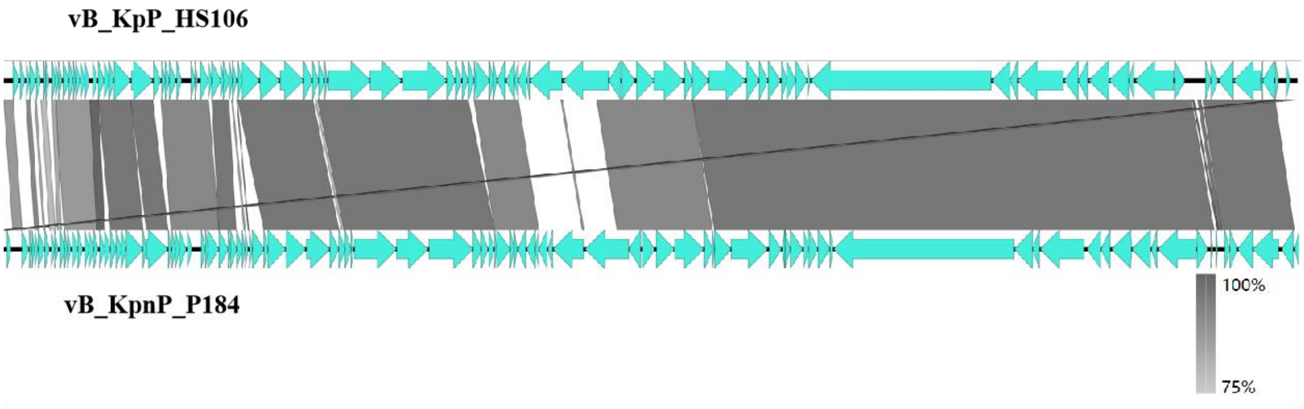

Supplementary Figure 1. Genome comparison of phage vB_KpP_HS106 and its reference phage vB_KpnP_P184, using BLASTn and visualization with EasyFig. Genome maps of phage vB_KpP_HS106 and phage vB_KpnP_P184 were presented as blue arrows. Regions of sequence similarity were connected by a gray-scale shaded area.
