# Supplementary material for: Isolation and characterization of novel bacteriophage vB_KpP_HS106 for Klebsiella pneumonia K2 and applications in foods
Source: Front Microbiol. 2023 Aug 16;14:1227147. doi: 10.3389/fmicb.2023.1227147 (PMC10466807; doi:10.3389/fmicb.2023.1227147)
Supplement: Supplementary file 2 [file Table_1.docx]

Supplementary Material

**Supplementary Table 1. The resistant phenotype of *K. pneumoniae* used in this study**

| **Strain** | **β -lactam** | **carbopenems** | **quinolones** | **tetracyclines** | **aminoglycoside** | **Chloramphenicol** | **sulfanilamide grous** | **Polypeptides (polymyxin E)** |
| --- | --- | --- | --- | --- | --- | --- | --- | --- |
| *K. pneumoniae* 001 | AMP, CFZ, CRO |  |  |  | GEN |  |  | COL |
| *K. pneumoniae* 002 | AMP, CFZ |  |  | DOX | GEN | CHL |  | COL |
| *K. pneumoniae* 006 | AMP, CFZ, CTX |  |  | DOX |  |  |  | COL |
| *K. pneumoniae* 011 | AMP, CFZ, CTX |  |  | DOX | GEN, VAN | CHL |  | COL |
| *K. pneumoniae* 015 | AMP,CFZ |  |  |  | GEN | CHL | SMZ | COL |
| *K. pneumoniae* 021 | AMP, CFZ, CTX, CAZ |  |  | DOX |  |  |  | COL |
| *K. pneumoniae* 023 | AMP, CFZ,CTX, CAZ | IPM |  | DOX |  |  | SMZ | COL |
| *K. pneumoniae* 031 | AMP, CFZ, CAZ | IPM |  | TET |  | CHL | SMZ | COL |
| *K. pneumoniae* 036 | AMP, CFZ, CTX, CAZ, CCL | IPM | CIP | TET, DOX | GEN |  | SMZ | COL |
| *K. pneumoniae* 037 | AMP, CFZ, CTX |  |  | TET, DOX | GEN | CHL | SMZ | COL |
| *K. pneumoniae* 042 | AMP, CFZ | IPM |  | DOX |  |  |  | COL |
| *K. pneumoniae* 045 | AMP, CFZ, CAZ |  |  |  | VAN | CHL | SMZ | COL |
| *K. pneumoniae* 048 | AMP, CFZ, CTX, CRO, CCL |  |  | TET, DOX | GEN |  | SMZ | COL |
| *K. pneumoniae* 052 | AMP, CFZ |  |  | DOX |  | CHL | SMZ | COL |
| *K. pneumoniae* 058 | AMP, CFZ , CTX, CAZ, CCL, CRO |  |  | DOX | GEN | CHL |  | COL |
| *K. pneumoniae* 059 | AMP, CFZ, CTX, CRO, CCL |  |  | TET, DOX | GEN | CHL | SMZ | COL |
| *K. pneumoniae* 060 | AMP, CFZ, CTX, CRO, CCL |  |  | TET, DOX |  |  |  | COL |
| *K. pneumoniae* 061 | AMP, CFZ , CTX, CAZ, CCL, CRO | IPM |  | TET, DOX | GEN | CHL | SMZ | COL |
| *K. pneumoniae* 062 | AMP, CFZ |  |  | DOX |  |  |  | COL |
| *K. pneumoniae* 071 | AMP, CFZ, CTX, CCL |  | CIP | TET, DOX | GEN | CHL | SMZ | COL |
| *K. pneumoniae* 072 | AMP, CFZ, CCL |  | CIP | TET, DOX | GEN |  | SMZ | COL |
| *K. pneumoniae* 074 | AMP, CFZ, CCL |  |  | TET, DOX | GEN |  | SMZ | COL |
| *K. pneumoniae* 077 | AMP, CFZ , CTX, CAZ, CCL, CRO | IPM |  | TET, DOX | GEN, VAN | CHL |  | COL |
| *K. pneumoniae* 102 | AMP, CFZ , CTX, CAZ, CCL, CRO |  |  | TET, DOX | GEN | CHL | SMZ | COL |
| *K. pneumoniae* 103 | AMP, CFZ , CTX, CAZ, CCL, CRO |  |  | TET, DOX |  | CHL |  | COL |
| *K. pneumoniae* 106 | AMP, CFZ , CTX, CAZ, CCL, CRO |  |  | TET |  | CHL | SMZ | COL |
| *K. pneumoniae* 111 | AMP, CFZ |  |  | TET | VAN | CHL |  | COL |
| *K. pneumoniae* 112 | AMP, CFZ , CTX, CAZ, CCL, |  |  | DOX | GEN | CHL | SMZ | COL |
| *K. pneumoniae* 204 | AMP, CFZ , CTX, CAZ, CCL, CRO | IPM | LML | TET, DOX | VAN | CHL |  | COL |
| *K. pneumoniae* 206 | AMP, CFZ , CTX, CAZ, CCL, CRO | IPM |  |  | VAN | CHL |  | COL |
| *K. pneumoniae* 208 | AMP, CFZ , CTX, CAZ, CCL, CRO |  |  | TET, DOX |  |  |  | COL |
| *K. pneumoniae* 211 | AMP, CFZ , CTX, CAZ, CCL, CRO | IPM |  | TET, DOX |  |  |  | COL |
| *K. pneumoniae* 304 | AMP, CFZ | IPM |  | TET, DOX | GEN | CHL | SMZ | COL |
| *K. pneumoniae* 308 | AMP, CFZ , CTX, CAZ, CCL, CRO |  | LML | TET, DOX | GEN, VAN |  | SMZ | COL |
| *K. pneumoniae* 309 | AMP, CFZ , CTX, CAZ, CCL, CRO |  |  | TET, DOX | GEN | CHL | SMZ | COL |
| *K. pneumoniae* 311 | AMP, CFZ , CTX, CAZ, CCL, CRO |  |  | TET, DOX | GEN, VAN | CHL | SMZ | COL |
| *K. pneumoniae* 313 | AMP, CFZ , CTX, CAZ, CCL, CRO | IPM |  | TET, DOX | GEN | CHL | SMZ | COL |
| *K. pneumoniae* 403 | AMP, CFZ , CTX, CAZ, CCL, CRO |  |  | TET, DOX |  | CHL |  | COL |
| *K. pneumoniae* 405 | AMP, CFZ , CTX, CAZ, CCL, CRO | IPM |  | TET, DOX | GEN |  |  | COL |
| *K. pneumoniae* 411 | AMP, CFZ , CTX, CAZ, CCL, CRO |  |  | TET, DOX | GEN, VAN |  | SMZ | COL |
| *K. pneumoniae* 413 | AMP, CFZ , CTX, CAZ, CCL, CRO |  |  | TET, DOX | GEN, VAN |  | SMZ | COL |

AMP: Ampicillin; CFZ: cefazolin ; CTX: cefotaxime ; CAZ: ceftazidime ; CCL: cefaclor; CRO: cefatriaxone; IPM: imipenem ; CIP: ciprofloxacin ; LML: lomefloxacin; DOC: doxycycline; TET: tetracycline; GEN: gentamicin; VAN: vancomycin; CHL: chloramghenico; SMZ: sulfamethoxazole; COL: polymyxins.
